# Connected at Heart? Socioeconomic Status and Physiological Linkage During Marital Interactions

**DOI:** 10.1093/geroni/igaf122.2102

**Published:** 2025-12-31

**Authors:** Tabea Meier, Aaron Geller, Kuan-Hua Chen, Claudia Haase

**Affiliations:** University of Zurich, Zürich, Switzerland; Northwestern University, Evanston, Illinois, United States; University of Nebraska Medical Center, Omaha, Nebraska, United States; Northwestern University, Evanston, Illinois, United States

## Abstract

Connections with our loved ones play a critical role in our health, and emotions during marital interactions in mid-life can predict the development of health problems in later life. Research has documented greater interdependence among people from lower socioeconomic status (SES) backgrounds, but little is known about whether greater interdependence also emerges at a physiological level in low-SES contexts. This laboratory-based study examined physiological linkage (i.e., coordinated changes in partners’ interbeat intervals, the time between two successive heart beats) in 48 married couples (96 spouses; M = 43.38, SD = 9.25, range: 21-68 years) from highly diverse SES and racialized backgrounds across two marital interaction contexts (conflict and pleasant conversations). We analyzed both in-phase (i.e., coordinated changes in the same direction) and anti-phase (i.e., coordinated changes in opposing directions) physiological linkage. The overall number of observations was N = 192 (48 couples with four repeated measures; i.e., in-phase and anti-phase linkage in conflict and pleasant conversations). Repeated measures analyses showed that, across both conversations, spouses from lower (vs. higher) SES backgrounds showed greater in-phase and lower anti-phase physiological linkage. That is, their heart rates were more likely to change in similar ways and less likely to change in opposite ways. These findings provide first evidence linking socioeconomic status—a key macro-level factor shaping resources, rank, and societal hierarchy—with physiological linkage in married couples. Viewed through the lens of physiological linkage as an amplifier of emotions, this study prompts further research on the risks and resources for healthy aging in couples from lower-SES backgrounds.

